# Endoscopic stricturotomy and strictureplasty for Crohn’s disease–related duodenal strictures

**DOI:** 10.1016/j.igie.2024.06.001

**Published:** 2024-06-21

**Authors:** Shanshan Wang, Nan Lan, Bo Shen

**Affiliations:** 1 Department of Gastroenterology and Hepatology, Hospital Universitario Puerta de Hierro de Majadahonda, Madrid, Spain; 2 Division of Digestive and Liver Diseases, Columbia University Irving Medical Center, New York, New York, USA; 3 The Global Integrated Center for Colorectal Surgery and Interventional IBD and Center for Inflammatory Bowel Diseases, Columbia University Irving Medical Center/NewYork Presbyterian Hospital, New York, New York, USA

## Abstract

**Background and Aims:**

Duodenal stricturing in Crohn’s disease is rare, and its management has been challenging. Fibrotic or mixed inflammatory/fibrotic primary strictures of Crohn’s disease do not respond well to medical therapy, eventually requiring endoscopic or surgical intervention. Endoscopic balloon dilation has been considered a middle-of-the-road strategy between medical and surgical approaches but is limited by its efficacy in primary stricture, recurrence, and frequent need for redilation. To address these issues, novel strategies, including endoscopic stricturotomy and strictureplasty utilizing electroincision, have been developed. This study aimed to analyze the effectiveness and safety of endoscopic electroincision therapy in Crohn’s disease–related duodenal strictures in a small cohort of consecutive patients.

**Methods:**

Data on patients diagnosed with Crohn’s disease and duodenal strictures who were treated with endoscopic electroincision were consecutively extracted from the interventional inflammatory bowel disease unit from December 2, 2019, to January 31 of 2024. All patients with anastomotic stricture were excluded from the study. The primary outcomes were surgery-free survival and postprocedural adverse events.

**Results:**

Eight endoscopic electroincision therapies were performed in 5 patients for Crohn’s disease–induced duodenal stricture. The study found a technical success rate of 88%, and a clinical response rate of 100%. The adverse event rate was unremarkable. The rate of endoscopic retreatment was 60%, with a minimum 6-month interval. In follow-up, no patient required surgical intervention.

**Conclusions:**

Both endoscopic stricturotomy and strictureplasty seem to be effective and safe therapeutic modalities for Crohn’s disease–associated duodenal stricture.

Crohn’s disease (CD) is a chronic, relapsing, inflammatory disease that can involve any portion of the GI tract.^1^ It is classified as nonstructuring/nonpenetrating, structuring, or penetrating, based on the disease behavior. The prevalence of duodenal involvement is estimated between .5% and 4%,^2^^,^^3^ although this rate might be underreported.^3^^,^^4^ Symptoms include epigastric pain, anorexia, nausea, vomiting, and weight loss. The main approaches for duodenal CD are medical and surgical interventions.^5^ Medical therapy, especially with biologic agents, can improve the inflammatory component of strictures ^6^^,^^7^ but is less efficacious for fibrotic strictures.^7^^,^^8^ Once patients present with obstructive symptoms, surgical intervention is often needed. However, surgery for CD, including duodenal strictures, is associated with risk for postoperative adverse events and recurrence.^9^^,^^10^ Less-invasive endoscopic therapy has gained momentum for the management of CD-associated strictures. Upper small-bowel strictures, treated with endoscopic balloon dilation (EBD),^11^^,^^12^ are also associated with frequent recurrence. Endoscopic stricturotomy (ESt) or endoscopic strictureplasty (ESTx) are widely performed in the distal bowel and provide a more effective treatment modality than EBD.^13^, ^14^, ^15^ However, to our knowledge, no data have been published regarding the upper GI tract. We report a case series of 5 patients who received endoscopic stricture therapy (ESt and/or ESTx) 8 times with success, constituting a promising modality to avoid or delay surgical interventions.

## Case series

Patient 1, a 49-year-old man with CD who was taking vedolizumab, presented with nausea, vomiting, and weight loss. In EGD, a benign-appearing and severe stenosis was found in the duodenal cap. EBD was attempted twice, without responses. A first insulated-tip (IT) stricturotomy was successfully performed, with clinical improvement in symptoms. Subsequent endoscopic retreatments were needed, using IT stricturotomy and strictureplasty with 2 clips, respectively, with a 12-month gap in between. In follow-up, the patient remained asymptomatic and gained weight.

Patient 2, a 27-year-old man with CD who was taking adalimumab, developed postprandial epigastric pain. EGD revealed an ulcerated, deformed, and moderate stricture in the duodenal cap. The patient was treated with IT stricturotomy combined with EBD to 12 mm. Six months later, a severe stricture was found in the previous location, not traversable by the gastroscope. A new EBD was performed to 15 mm, making the stenosis passable after treatment. However, symptoms recurred over time, and colorectal surgery was consulted.

**Figure 1:**
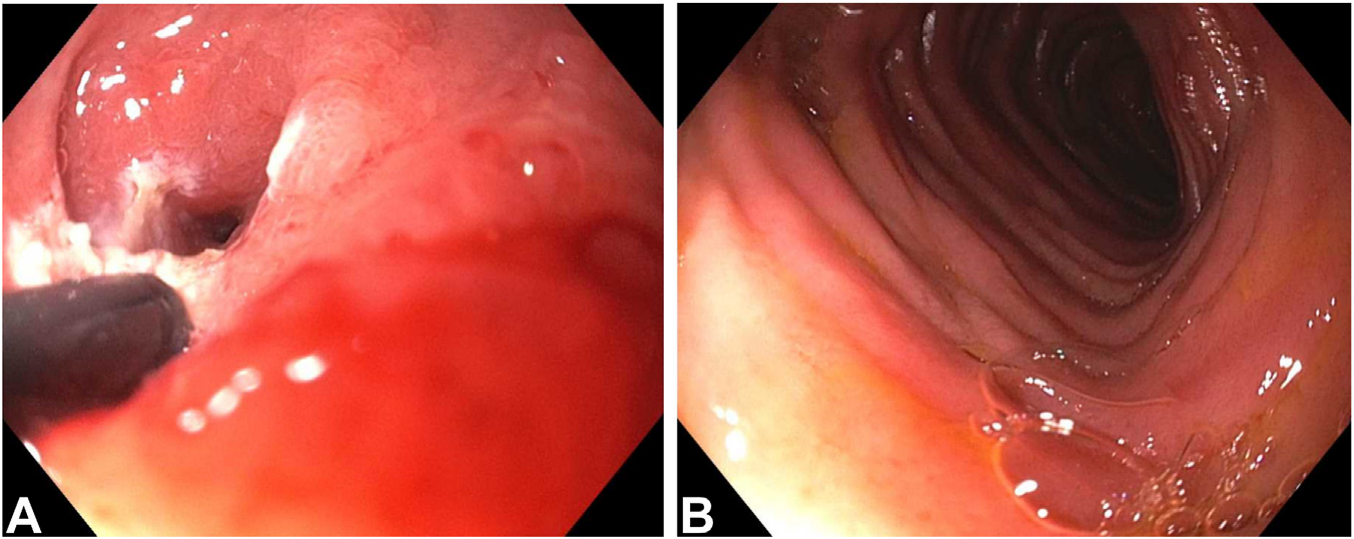
Endoscopic stricturotomy of a duodenal stricture in Crohn’s disease. **A,** At the electroexcitation. **B,** Postprocedure appearance.

Patient 3, a 52-year-old man with CD who was taking ustekinumab and who had a duodenal stricture previously treated with EBD, was asymptomatic. In EGD, a benign-appearing and severe stenosis was observed in the distal duodenal bulb. IT stricturotomy (Fig. 1) was performed without adverse events. The patient remained asymptomatic and gained weight during follow-up.

Patient 4, a 44-year-old man with small- and large-bowel CD who was taking ustekinumab and who had undergone ileocecal resection, presented with left abdominal pain. A history of ventral hernia repaired with mesh was noted. In EGD, a benign-appearing, nonulcerated, moderate duodenum cap stricture was found and successfully treated with IT stricturotomy. After the procedure, the patient’s pain control improved, and he maintained weight. Six months later, a severe stricture reappeared in the same site and a not previously described pyloric stricture was found. IT stricturotomy was successfully performed at both locations.

Patient 5, a 37-year-old woman with CD, ileocecal resection, and anal stenosis, developed bloating and postprandial abdominal pain. The patient had failed to improve with multiple biologic agents and was currently taking risankizumab. Gastric granuloma was shown in the previous examination. In EGD, a benign-appearing, nonulcerated, severe stenosis was found in the duodenum cap. IT stricturotomy was completed, with the stricture traversable afterward. No recurrence of duodenal stricture was recorded in subsequent endoscopies.

Clinical data and treatment outcomes are listed in Table 1 and Table 2, respectively.

**Table 1:** Demographic and clinical data and treatment

Variables	Case 1			Case 2		Case 3	Case 4		Case 5
Age at first endoscopic therapy, y	49			27		52	44		37
Sex	Male			Male		Male	Male		Female
Phenotype of Crohn’s disease based on the Montreal classification	L1+L4B2			L1+L4B2		L2+L4B2	L2+L4B2p		L3+ L4B2p
Previous attempts of EBD	Yes	Yes	Yes	No	Yes	Yes	Yes	Yes	No
Previous surgery for duodenal stricture	No	No	No	No	No	No	No	No	No
Concurrent biologics/small molecule agents	Vedolizumab			Adalimumab		Ustekinumab	Ustekinumab + methotrexate		Risankizumab
Location of stricture	D2			cap		cap	cap		cap
Length of stricture, cm	1	1.5	1.5	2.5	Angulated	2	1	1.5	1
Number of stricture	1	1	1	1	1	1	1	2	1
Severity of stricture (severe = not traversable to gastroscope; moderate = traversable but passage with resistance)	Severe	Severe	Severe	Moderate	Severe	Severe	Severe	Moderate	Severe
Inflammatory/mixed/fibrotic	Fibrotic	Fibrotic	Fibrotic	Fibrotic	Fibrotic	Fibrotic	Mixed	Fibrotic	Fibrotic
Stricturotomy/strictureplasty outcome	ESt	ESt	ESTx	ESt+EBD	EBD	ESt	ESt	ESt	ESt
Improvement of symptoms	Yes	Yes	Yes	Yes	Yes	Yes	Yes	Yes	Yes
Technical success	Yes	Yes	No	Yes	Yes	Yes	Yes	Yes	Yes
Requirement endoscopic reintervention	Yes	Yes	–	Yes	–	–	Yes	–	No
Interval between index stricturotomy/strictureplasty and subsequent endoscopy treatment, mo	12	12	–	6	–	–	6	–	–
Escalation of medication for duodenal stricture	No	Yes	No	No	No	No	No	No	Yes
Requirement of surgical intervention for failure of stricturotomy	No	No	No	No	No	No	No	No	No
Procedure-associated adverse events (bleeding/perforation/ileus)	No	No	No	No	No	No	No	No	No∗

*EBD*, Endoscopic balloon dilation; *D2*, second portion of the duodenum; *ESt*, endoscopic stricturotomy; *ESTx*, endoscopic strictureplasty.

∗ The patient experienced nausea after the procedure, but no bleeding, perforation, or ileus was observed in this patient.

**Table 2:** Treatment outcomes

Short-term efficacy	Rate	Long-term efficacy	Rate
Technical success (the ability to pass the scope beyond stricture after interventions)	88% (7/8)	Requirement for endoscopic retreatment	60% (3/5)
Clinical response (relief of pain or obstructive symptoms)	100% (8/8)	Requirement for surgical interventions	0% (0/8)
Adverse events (procedure-related intense abdominal pain, bleeding, and perforation)	0% (0/8)		

## Discussion

We present a case series of 8 endoscopic stricture therapies (ESt or ESTx) performed on 5 patients for CD-related duodenal strictures. Stricturotomy was conducted through electroincision in a radial, circumferential, or horizontal way to widen the narrowed lumen. The goal for immediate technical success was to widen the lumen, allowing for easy passage of a gastroscopy. Patients with short strictures and a radial or horizontal cut had strictureplasty with endoclips placed in the electroincised areas to maintain the patency of the lumen. IT knives were used in the setting of ERCP ENDO CUT 1 (effect 3, duration 2, and interval 3; Erbe USA, Marietta, Ga, USA).

Most of the strictures were short (1-2.5 cm), primary (from underlying disease), and severe, which means not traversable to an EGD scope. Our series found a technical success rate of 88% and a clinical response rate of 100%. In one procedure, the stricture was not traversable after treatment due to looping scope, but the patient stated clinical relief. The adverse event rate was unremarkable. All patients were discharged on the same day the procedure was performed. Patient 5, without a history of coagulation disorder or any use of anticoagulation treatment, presented with duodenal hemorrhage 3 months later that required endoscopic hemostasia, unlikely related to the procedure. Thus far, none of our patients have had surgery; 1 (Patient 2) is pending surgery consult. Retreatment (with ESt, ESTx, or EBD) was necessary for 3 of 5 patients, with a minimum 6-month interval. The other 2 (Patients 3 and 5) have not shown recurrent stricture, considering one of the ESt procedures (Patient 3) was performed 3 months ago. The ESt appears to have a similar technical and clinical success rate between the index and subsequent procedure in the same patient.

For CD strictures, the main endoscopic treatment modalities are EBD, ESt, ESTx, and endoscopic stenting.^16^ For a considerable time, EBD has been presented as a middle-of-the-road strategy between medical and surgical approaches. Previous studies have proved it to be an effective (success rate of 92.5%-93%) and safe (3%-4% adverse event rate per procedure, mainly perforations and bleedings) therapy.^8^^,^^17^ The major limitation of EBD is its long-term efficacy. About two-thirds of patients required redilation in a mean time of 2.2 months, and one-third ended up needing surgery.^8^ Compared with EBD, ESt was found to be more effective and safer, with a lower perforation rate, likely due to the technical differences. ESt allows targeting with precision while EBD exerts an equal blind, radial force on an asymmetric stenosis.^18^ A higher risk for late-onset bleeding for ESt was noted (around 3%).^19^ In our experience, this can be reduced by self-assembling peptide hemostatic gel (PuraStat, 3-D Matrix Medical Technology, Tokyo, Japan), widely used in routine practice. Endoscopic spray of hemostatic gel is used for both the treatment and prevention of procedure-associated bleeding in almost all patients with IBD who underwent endoscopic therapy. Before its availability, we used 50% glucose spray for the treatment and prophylaxis of bleeding.

Endoscopic stenting remained anecdotal in the upper GI tract.^20^ Surgical options for CD duodenal strictures include resection, intestinal bypass, and strictureplasty.^5^^,^^21^ In a comparative study on endoscopic ESt versus surgical resection for ileum strictures, ESt seemed to have a surgery-free survival comparable to that of surgery, with a lower adverse event rate.^15^ No comparison between ESt and surgical intervention for foregut stricture has been made thus far. Nonetheless, surgical resection plays a relevant role for strictures not amendable or failed to endoscopic treatment.

We report the first case series with the serial treatment of ESt and ESTx for CD-related duodenal strictures. Our results suggest that, for duodenal strictures in CD, endoscopic electroincision is an effective and safe endoscopic approach in experienced hands, with a slightly better long-term efficacy than EBD. For fibrotic strictures, ESt and ESTx seem to be more effective than EBD. However, for inflammatory strictures, EBD should be considered the first choice, akin to CD-related strictures in the distal bowel. The clips can be applied after ESt for selected patients, especially in refractory or angulated strictures. Patients with short strictures <2 cm who undergo horizontal or radial electroincision usually benefit more from ESTx than ESt. Nevertheless, prospective studies with a larger number of patients and long-term follow-up are warranted to clarify indications of these novel managements.

In conclusion, ESt and ESTx are emerging endoscopic therapies that seem to be efficacious and minimally invasive alternatives for CD-associated duodenal stricture.

## Disclosure

All authors disclosed no financial relationships.
